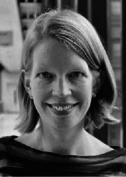# Targeting TRIM Proteins: A Quest towards Drugging an Emerging Protein Class

**DOI:** 10.1002/cbic.202000787

**Published:** 2021-03-18

**Authors:** Francesca D'Amico, Rishov Mukhopadhyay, Huib Ovaa, Monique P. C. Mulder

**Affiliations:** ^1^ Oncode Institute and Department of Cell and Chemical Biology Leiden University Medical Center (LUMC) Einthovenweg 20 2333 ZC Leiden The Netherlands

**Keywords:** high-throughput screening, inhibitors, ligases

## Abstract

The ubiquitylation machinery regulates several fundamental biological processes from protein homeostasis to a wide variety of cellular signaling pathways. As a consequence, its dysregulation is linked to diseases including cancer, neurodegeneration, and autoimmunity. With this review, we aim to highlight the therapeutic potential of targeting E3 ligases, with a special focus on an emerging class of RING ligases, named tri‐partite motif (TRIM) proteins, whose role as targets for drug development is currently gaining pharmaceutical attention. TRIM proteins exert their catalytic activity as scaffolds involved in many protein–protein interactions, whose multidomains and adapter‐like nature make their druggability very challenging. Herein, we give an overview of the current understanding of this class of single polypeptide RING E3 ligases and discuss potential targeting options.

## Graphical abstract

**It can be hard to get TRIM**: TRIM proteins, the largest subfamily of single polypeptide RING E3 ligases, are currently gaining attention as targets for drug development. However, developing effective targeting options for TRIMs is challenged by their multidomain scaffolding nature correlating with multifunctional biological roles. Herein, we highlight our current understanding of TRIMs and discuss potential targeting options.

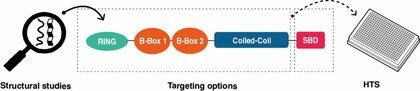

## 1 Introduction

Ubiquitylation is a highly complex post‐translational modification (PTM) that is stringently conserved in all eukaryotic cells.^[1]^ It is a crucial cellular process targeting a substrate protein with a specific pattern of ubiquitin (Ub) molecules, thereby determining its cellular fate.^[2]^ Physiologically, ubiquitylation regulates protein degradation through the ubiquitin proteasome system (UPS) and modulates a wide variety of cellular signaling pathways, including cell differentiation and proliferation, apoptosis, DNA damage response, protein localization and trafficking, autophagy, and endocytosis.^[3]^ In particular, the ubiquitylation machinery is linked to the regulation of the stability and activity of tumor suppressor or oncogene products,^[4]^ to the ability of cells to degrade toxic protein aggregates,^[5]^ and to the activation and aberration of specific inflammatory pathways.^[6]^ Consequently, dysregulation of this system can lead to several pathological conditions, including cancer onset and progression, neurodegeneration, and autoimmunity. Therefore, targeting the UPS has tremendous potential for intervention on multiple pathologies and is considered a privileged pharmacological target for drug development.^[7]^

A huge number of possible combinations of ubiquitin patterns decorating the substrate protein can be obtained, generating a complex and dynamic “ubiquitin code“. This code is made up of “writers” (E1–E2–E3 enzymes) responsible for ubiquitylation that can be reversed or modified by “erasers/editors” deubiquitinases (DUBs). Finally, “readers”, carrying ubiquitin binding domains (UBDs), are able to recognize with specific affinity ubiquitylated substrates and to interpret the resulting Ub chain patterns built on the substrate lysine.^[8]^ The complexity of the system is increased by considering that substrates can be modified by Ub not only on a single Lys residue (mono‐ubiquitylation), but also on multiple Lys residues (multi‐monoubiquitylation). Additionally, alternative Ub modifications have been reported, for example, conjugation via a peptide bond to the N‐terminal amino group of the substrate^[9]^ or via thio‐ or oxy‐ester bonds to cysteine or serine/threonine residues, respectively.^[10]^ Moreover, Ub can be post‐translationally modified itself by ubiquitylation on its seven lysine residues (K6, K11, K27, K29, K33, K48, K63) and its N‐terminal methionine leading to the formation of polyubiquitin chains. According to the number, linkage and sites of ubiquitylation, different chain architectures can be obtained (homogeneous, alternated, branched).^[8]^ One more layer of complexity is given by the possibility of Ub to undergo different types of PTMs, such as ribosylation, phosphorylation, acetylation and conjugation to ubiquitin‐like proteins (UBLs).^[11]^

**Figure 1:**
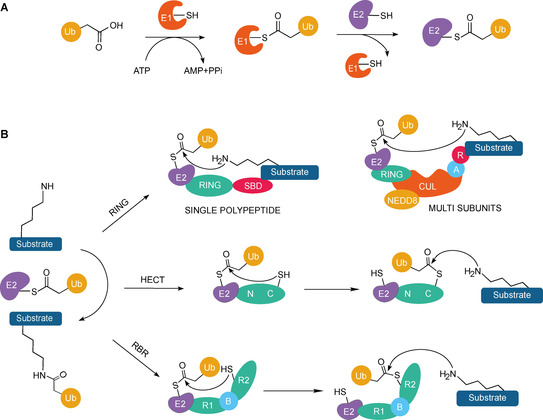
Mechanism of ubiquitin transfer. A) Ub forms a thioester intermediate with the E1 and is then transferred to the E2 through a trans‐thiolation reaction. B) Next, E3 ligases (RING, HECT or RBR) catalyze the transfer of Ub onto the substrate directly or by intermediacy of a Ub‐thioester. SBD: substrate binding domain; CUL: cullin protein; A: adaptor; R: substrate‐receptor; C: C‐terminal domain, N: N‐terminal domain; R1: RING domain 1, B: in‐between domain, R2: RING domain 2.

The ubiquitin code is written by the finely tuned and combined action of three types of enzymes, named E1 ubiquitin activating enzyme, E2 ubiquitin conjugating enzyme, and E3 ubiquitin ligase. By consuming ATP, E1 forms a high‐energy thioester intermediate on its active‐site Cys with Ub that is then transferred on the E2 Cys active site through a trans‐thiolation reaction^[8]^ (Figure 1A). At this stage, E3 ligases play a crucial role in substrate recruitment and specificity, catalyzing the transfer of Ub onto the substrate directly or by the intermediacy of a ubiquitylated E3, depending on the nature of the ligase involved (Figure 1B). Three general classes of E3 s based on mechanism and topology of Ub transfer have been identified: really interesting new gene (RING), homologous to E6AP C terminus (HECT) and ring‐between‐RING (RBR). HECT and RBR ligases become ubiquitylated on their Cys active site via trans‐thiolation reaction with the E2‐Ub conjugate, before releasing Ub onto the substrate. RING ligases, instead, act as scaffolds accommodating concomitantly the E2‐Ub thioester and the substrate to catalyze direct transfer of Ub onto the target protein.^[12]^

Addressing the activity of E3 s to unveil new therapeutic strategies, relies on the identification of an effective targeting mode depending on the nature of the ligase involved. Whilst HECT and RBR detain an active‐site Cys that can be exploited for the design of covalent‐based inhibitors and probes,^[13]^ RING ligases are devoid of such a unique catalytic site. Additionally, RING ligases can be further classified as “multi‐subunits” and “single polypeptide” RING E3 s (Figure 1B). The former are exemplified by the cullin‐RING ligases superfamily (CRL), whose biological function is achieved by binding to additional partners or adaptors, offering multiple targeting sites.^[14]^ The latter are described as single polypeptide RING E3 with peculiar regulatory events of activation and catalysis that are yet to be elucidated.^[15]^

**Figure 2:**
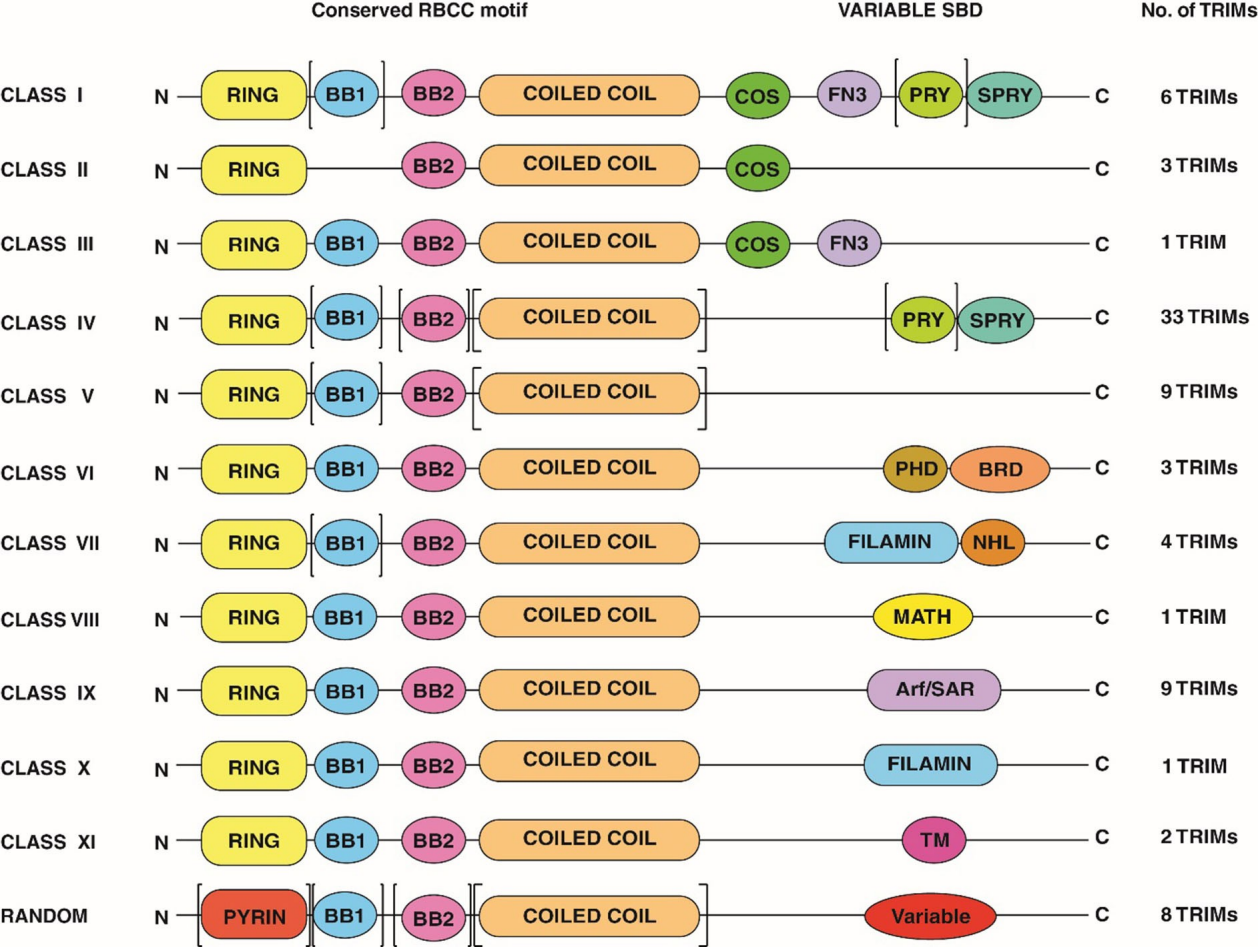
Classification of TRIM/RBCC proteins. The N‐terminal domain (N) or RBCC motif is mostly conserved in TRIM family members and includes RING, BB1 (B‐box 1), BB2 (B‐box 2), and CC (coiled‐coil domain). A variable C‐terminal domain (C) classifies TRIMs into 12 different classes and includes COS (COS box motif), FN3 (FibroNectin type III motif), PRY, SPRY (SPla and the RYanodine receptor), PHD (Plant Homeodomain), BRD (bromo domain), FILAMIN, NHL (NCL‐ 1/HT2A/LIN‐41), MATH (meprin and TRAF homology domain), ARF (ADP ribosylation factor)/SAR, TM (transmembrane motif), and a variable domain. The presence of certain domains can vary even among members of the same class, as indicated by brackets.

Tripartite motif proteins (TRIMs) represent the largest subfamily of single polypeptide RING E3 ligases, including approximately 80 members in humans (Figure 2), that have been reported to catalyze direct transfer of Ub,^[16]^ SUMO^[17]^ or ISG15^[18]^ onto the substrate protein. However, not all TRIM proteins have been functionally characterized so far. Dysregulation of TRIM ligases has been linked to a variety of pathological conditions, as summarized in Table 1.^[19]^ Therefore, their importance as targets for drug discovery is gradually gaining attention. In this review, we provide an overview of the current understanding of this emerging class of single polypeptide RING E3 ligases. Here, we highlight challenges in their druggability and discuss potential targeting options.

**Table 1:** Summary of the TRIM ligases involved in human diseases.

Type of disease	Disease	TRIM gene involved
developmental and rare diseases	limb girdle muscular dystrophy type 2H^[19]^	TRIM32^[25]^
	ataxia telangiectasia^[19]^	TRIM29, TRIM8^[19]^
	Smith Lemli Opitz syndrome^[19]^	TRIM29^[19]^
	Bardet‐Biedl syndrome^[19]^	TRIM32^[26]^
	Williams Beuren syndrome^[19]^	TRIM50^[27]^
neuropsychiatric diseases	multiple sclerosis^[19]^	TRIM5, TRIM10, TRIM15, TRIM20, TRIM26, TRIM39, TRIM40
	Alzheimer's disease[^19^, ^28^]	TRIM20, TRIM2
	schizophrenia[^19^, ^29^]	TRIM24, TRIM32, TRIM3, TRIM19, TRIM26, TRIM70
	Parkinson's disease[^19^, ^30^]	TRIM9
metabolic and cardiac disorders	obesity[^19^, ^31^]	TRIM3, TRIM23, TRIM25
	muscular atrophy and cardiac myopathies[^19^, ^32^, ^33^]	TRIM63, TRIM76, TRIM72
	diabetes^[19]^	TRIM72, TRIM31
	diabetic nephropathy[^19^, ^34^]	TRIM13
infectious diseases	viral infections like HIV, HCV, SARS‐COV, MERS‐COV, etc.^[35]^	TRIM25, TRIM5α, TRIM28, TRIM33, TRIM24
	*Salmonella typhimurium* infection^[36]^	TRIM56, TRIM65
cancers	prostate cancer[^37^, ^38^]	TRIM24, TRIM25,^[39]^ TRIM13, TRIM8, TRIM44, TRIM47
	breast cancer	TRIM28,[^40^, ^41^, ^42^] TRIM25,[^43^, ^44^] TRIM11,[^45^, ^46^] TRIM14[^46^, ^47^]
	brain tumor[^37^, ^38^]	TRIM33,^[48]^ TRIM45
	gastric cancer[^37^, ^38^]	TRIM29, TRIM25
	pro‐myelocytic leukemia[^37^, ^38^]	TRIM19
	colorectal cancer[^37^, ^38^]	TRIM65, TRIM25,^[49]^ TRIM52, TRIM59, TRIM28,^[50]^ TRIM29,^[50]^ TRIM23,^[50]^ TRIM24,^[50]^ TRIM25^[50]^
	neuroblastoma[^37^, ^38^]	TRIM59
	squamous cell carcinoma[^37^, ^38^]	TRIM28,^[51]^ TRIM29
	osteosarcoma and epithelial mesenchymal transition[^37^, ^38^]	TRIM14, TRIM16, TRIM28,^[52]^ TRIM66^[53]^
	intraductal carcinoma[^37^, ^38^]	TRIM27, TRIM28^[54]^
	non‐small‐cell lung carcinoma[^37^, ^38^]	TRIM59, TRIM67,^[55]^ TRIM71
	hepatocellular carcinoma[^37^, ^38^]	TRIM31, TRIM26
	bladder urothelial carcinoma[^37^, ^38^]	TRIM29,^[56]^ TRIM65
	glioblastoma, chronic lymphocytic leukemia^[57]^	TRIM 8, TRIM24,^[58]^ TRIM28 (in Glioma)^[59]^

## 2 Emerging Drug Development Strategies Targeting the Ubiquitin Machinery

Considering the impact of ubiquitylation on the regulation of a vast array of fundamental biological processes, all enzymes involved in the ubiquitylation machinery are emerging as potential targets for drug discovery.^[7]^ However, as E3 ligases play a crucial role in substrate recruitment and target specificity, they have gained more pharmaceutical attention.^[20]^

The possibility of interfering with ubiquitin signaling for therapeutic purposes was in first instance evaluated through the development of peptide‐based inhibitors that directly target the proteasome, preventing degradation of ubiquitylated proteins.^[21]^ Structurally, these inhibitors contain an electrophilic site attached to a linear or cyclic peptide chain that mimics the substrate protein.^[21]^ Their binding is based on the interaction of this electrophile with key nucleophilic Thr residues in the catalytic β1, β3, β5 subunits of the proteasome.^[22]^ Among these inhibitors, Bortezomib (Velcade®) and Carfilzomib (Kyprolis®) are the only two currently approved drugs that target the UPS and they are clinically used for the treatment of multiple myeloma.^[23]^ Nevertheless, targeting of the proteasome itself can negatively influence a vast array of protein networks as it results in an overall increase of protein levels.^[24]^ An alternative way of targeting the UPS is by inhibition of the ubiquitylating enzymes.

In humans, there are two known E1‐activating enzymes^[60]^ – UBA1, UBA6 (MOP4) – and around 40 different E2‐conjugating enzymes.^[61]^ E1 inhibitors have been extensively developed and the topic has been previously reviewed.^[62]^ Targeting ubiquitylation at the E1 level globally disrupts the UPS, similarly to the effects provoked by directly targeting of the proteasome. Conversely, inhibition at the E2 level would confer higher target specificity. However, few options for E2 inhibition have been reported to date: CC0651 that allosterically binds hCdc34 preventing p27^Kip1^ ubiquitylation, thus inhibiting cell proliferation;^[63]^ compound TZ9, developed by the use of a virtual screening approach, that competitively binds the catalytic site of Rad6, essential for post‐replication DNA repair;^[64]^ NSC697923 and BAY 11‐7082 that both inhibit Ubc13 by covalent adduct formation through a Michael addition at the Cys active site, thus inhibiting DNA damage and NF‐κB signaling in human cells.^[65]^

As the regulation of Ub signaling is hierarchical with more than 600 E3 ligases reported to date, playing a crucial role in substrate recruitment and specificity, targeting the activity of E3 s represents the most powerful strategy to manipulate the Ub system. Despite the importance of this class of enzymes, their activity and related regulatory mechanisms remain enigmatic for most, especially in the case of “non‐Cys active site’’ ligases. In particular, RING ligases function as scaffolds bringing other proteins in close proximity and how this spatial arrangement promotes the isopeptide bond formation still remains unknown.^[15]^ Notoriously, these scaffolding non‐enzymatic proteins, lacking well‐defined catalytic pockets, have for long been considered outside of the druggable space of cellular targets. However, after the discovery of immunomodulatory drugs thalidomide, pomalidomide and lenalidomide which bind cereblon (CRBN), the substrate‐receptor component of a DCX (DDB1‐CUL4‐X‐box) E3 protein ligase complex,^[66]^ the activity and the structure of multi‐subunit cullin‐RING ligases could be characterized, allowing the identification of structural protein domains suitable for the development of inhibitors.^[14]^ To date, both peptidic and small‐molecule inhibitors have been designed for Von‐Hippel‐Lindau protein (VHL), the substrate‐receptor region of the multi‐subunit E3 CUL2^VHL^;^[67]^ Mouse double minute 2 homologue (MDM2), which plays a crucial role in p53 stabilization,^[68]^ and Inhibitor of apoptosis proteins (IAPs), key regulator of cell survival.^[69]^ All represent illustrative examples of effective targeting strategies directed on RING ligases and the topic has been exquisitely reviewed recently.^[70]^

Targeting the ligase activity of TRIM proteins has recently emerged as a promising therapeutic strategy. For instance, in the treatment of cancer, as members of this family can modulate the stability and function of important regulators of carcinogenesis through their ubiquitylation activity, driving cell cycle progression or apoptosis, proliferation or differentiation, respectively.^[56]^ Additionally, there are several examples of TRIM proteins that function as nuclear receptors and transcription co‐activators/co‐repressors of oncogenic pathways.^[56]^ Importantly, some TRIMs are encoded by genes involved in specific cancer‐related chromosomal re‐arrangements. An illustrative example is offered by TRIM19 that is encoded by the promyelocytic leukemia (*PML*) gene, responsible for acute promyelocytic leukemia (APL).^[71]^ The wide role of TRIMs in various human ailments is summarized in Table 1.

## 3 TRIpartite Motif Proteins: Structural Determinants

Despite the emerging role of TRIM ligases in diseases and their importance as targets for drug discovery, addressing the activity of this class of E3 s comes with several challenges. TRIM proteins, as RING ligases, belong to the largest class of E3 s that are devoid of a catalytic Cys; therefore, the identification of an effective targeting mode cannot rely on abolishment of enzyme activity through direct covalent inhibition of the catalytic (nucleophilic) site. Conversely, manipulating the activity of TRIM proteins, requires a detailed analysis of their multidomain nature. Identification of potentially druggable structural motifs and characterization of their function will ultimately help to define valuable targeting options within this subclass of proteins.

TRIM proteins, also referred as RBCC (RING, B‐Box 1/2, coiled‐coil) proteins, are characterized by the presence of a conserved N terminus which includes one RING domain, one or two B‐Box domains and a coiled‐coil domain, representing the hallmark of all family members. A variable C‐terminal substrate binding domain (SBD) has been identified to convey substrate recruitment and specificity.^[72]^ Based on the presence and arrangement of these domains, TRIMs have been grouped into 12 different classes (Figure 2).

### 3.1 RING domain

**Figure 3:**
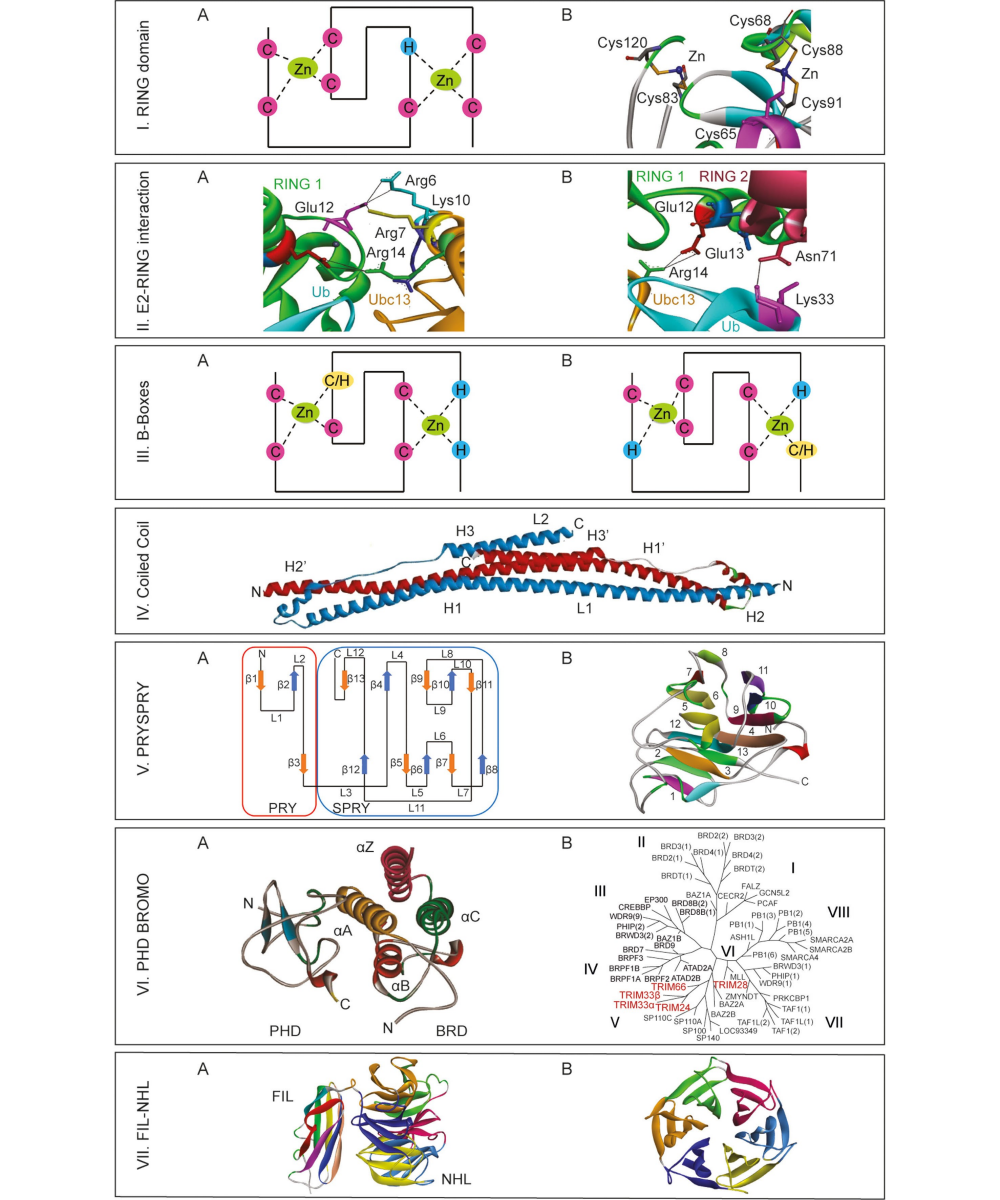
TRIM structural determinants. **I**. RING domain. A) Schematic representation of the RING domain: the coordination of zinc ions is tetrahedral and is based upon the position and the number of Cys and His residues. B) TRIM28 RING domain (PDB ID: 6I9H) coordination of the zinc ions. **II**. E2‐RING interaction. A) TRIM21^RING^ and the isopeptide‐linked Ube2N (Ubc13)‐Ub (PDB ID: 6S53): Glu12 and Asp21 interact with Ube2N via its positively charged residues Arg6, Arg7, Lys10 and Arg14, present on helix 1 of Ube2N, facing the TRIM^RING^ surface. The number of aromatic side chains at the E2 : E3^RING^ interface mediates hydrophobic and conserved H‐bond interactions. B) The TRIM^RING^ protomer interacts with Ub by forming H‐bonds between its Asn71 and Lys33. **III**. B‐Boxes. Schematic representations of A) B‐box 1 and B) B‐box 2. **IV**. Coiled Coil domain. TRIM25 coiled‐coil domain (PDB ID: 4LTB): within the symmetrical dimer each subunit shows a hairpin‐like folding forming long (L1) and short arms (L2). **V**. PRYSPRY domain. A) Schematic representation of the PRYSPRY domain, consisting of a PRY domain at its the N terminus, including 3 antiparallel β‐strands, and a SPRY domain that forms the remaining 10 strands towards its C terminus. B) PRYSPRY domain of TRIM5α (PDB ID: 2FBE): a compact module folded to acquire a jellyroll β‐sandwich tertiary conformation. **VI**. PHD‐BROMO domain. A) TRIM24 PHD‐BRD (PDB ID: 5H1U): The PHD is a zinc finger where C4HC3 cross braces to form antiparallel‐β‐sheets. The BRD folds with four left‐handed α‐helices (αZ, αA, αB, and αC) that form a large central cavity. The loop regions (ZA and BC loops) constitute the rim of the acetyl‐Lys binding pocket. B) Phylogenetic representation of the human BRD family, including 8 clusters (I‐VIII).^[112]^ TRIM proteins are non‐BET BRDs: TRIM24, TRIM33, and TRIM66 belong to subfamily V; TRIM28 is member of subfamily VI. **VII**. FILAMIN‐NHL domain (PDB ID: 6FPT). A) In TRIM proteins, the NHL domain is associated with the Filamin‐type immunoglobulin domain (FN3) to form a compact structure. The overall tertiary conformation of the NHL domain results in a β‐propeller barrel with a canonical toroidal shape.^[116]^ B) The NHL domain is present as multiple repetition units arranged in four to six symmetrical blade‐shaped β‐sheets.[^28^, ^72^]

The N terminus RING domain is the “catalytic center” of TRIMs, responsible for the engagement of the thioester linked E2‐Ub conjugate. Structurally, RING domains are zinc‐finger proteins with eight chelating residues (Cys or His) tetrahedrally coordinating two zinc ions (Figure 3 I A). Initially, these zinc finger domains were identified as A‐box preceding the B‐boxes and thought to consist of 60–80 amino acids with eight zinc‐binding Cys. Later, also His was identified to coordinate the zinc ions and A‐box was renamed the RING domain. This His and Cys arrangement is well conserved across species and is structurally significant to obtain active tertiary conformation (Figure 3 I B).^[72]^

The active conformation of TRIM proteins appears to be variable among family members. In some cases, dimerization of the RING domain is required for activity, as it has been reported for TRIM69 and TRIM32.[^73^, ^74^] RING homo‐dimerization is mediated by two short helices identified by the N‐ and C‐terminal regions adjacent to the core of the RING domain that are predicted to form a four‐helix bundle in the dimeric RING structure.^[73]^ However, the propensity to dimerize can be affected by several factors, like binding to the E2‐Ub conjugate,^[73]^ or additional cellular events (phosphorylation, RNA binding). For example, TRIM25 has been detected as a monomer in solution, but forms dimers in presence of the E2‐Ub conjugate.^[73]^ This self‐association is expected to be controlled by the coiled‐coil domain, although the precise mechanism is yet to be elucidated.^[73]^ Koliopoulos et al. described an intramolecular RING dimerization of TRIM25 in which the RING and the B‐box domains bind back across the coiled‐coil,^[73]^ while the dimerization model reported by Sanchez et al. describes an antiparallel TRIM25 dimer which positions the two RING domains on opposite ends of a 170‐Å‐long rod.^[75]^

Other members of the TRIM family like Class VI (TRIM24, 28 and 33) exist as monomers, since they lack the structural elements required for dimerization of the RING domain.^[76]^ For example, the crystal structure of TRIM28^RING^ shows that, despite conservation of the overall RING domain fold, the regions adjacent to the core RING are flexible and largely unstructured, in contrast to what has been observed for dimeric TRIMs.^[77]^ Intriguingly, the isolated RING domains of these class VI members do not show ubiquitylation activity as they are unable to promote ubiquitin discharge from E2‐Ub conjugates.^[76]^ Nevertheless, E3 ligase activity of Class VI TRIMs has been reported in cellular assays.[^78^, ^79^] This activity could be explained by the presence of cellular binding partners, post‐translational modifications or alternatively they might constitute inactive RING ligases that require heterodimerization with active RING partners for physiological activity.^[76]^

Furthermore, there are relevant examples of TRIM proteins undergoing higher order oligomerization prior activation, as illustrated by TRIM5α and TRIM19, whose RING active states are trimeric and tetrameric, respectively.[^80^, ^81^]

The above‐mentioned examples emphasize the importance of investigating structural features and active conformations of TRIMs to understand if their difference relate to mechanistic variation across family members and if this aspect can be explored for targeting. Interestingly, ligase activity of TRIMs might not only be dependent upon the RING domain as discussed in Section 3.3 in detail.

### 3.2 E2‐RING interaction

Recent studies directed on E3:E2‐Ub complexes have revealed new insights towards the interplay between E2‐Ub conjugates and RING E3 s that is deemed to be a general requirement for all RING E3 s, including TRIMs. E2‐Ub thioesters have a flexible topology; they can assume different configurations that are altered by E3 binding. In detail, the E2/E3 recognition event reduces E2‐Ub dynamics and promotes the stabilization of a closed or “folded back” conformation,^[82]^ placing the Ub moiety in close proximity to the substrate and priming the thioester intermediate for nucleophilic attack by the substrate Lys (Figure 1).^[83]^ In line with this observation, current available structural information on TRIM21 reveals that negatively charged residues on the RING domain of the TRIM undergo ionic interactions with both E2 and Ub to stabilize the complex^[84]^ (Figure 3 II A, B). The identification of this conserved binding mode across members of the TRIM family could define conserved residues and motifs to be exploited for targeting.

### 3.3 B‐Box domains

Following the RING domain, TRIM proteins contain one or two B‐Box domains (Figure 3 III A, B). The majority of TRIMs own two B‐Box domains in a conserved sequence arrangement B‐Box 1‐ B‐Box 2; however, TRIM proteins can contain only one B‐Box domain (Figure 2).^[73]^ The biological roles associated to the B‐Box domains in TRIMs are not yet fully understood. However, their function could be crucial in regulating TRIM activity. For example, it was recently observed that TRIM21 B‐Box 2 domain auto‐inhibits E3 activity by occluding the E2‐binding surface of the RING domain.[^85^, ^86^] Additionally, there is good evidence that B‐Boxes can drive higher‐order oligomerization as reported for TRIM5α and TRIM19. Here, B‐box residues (e. g., Trp, Phe and Leu) mediate hydrophobic interaction facilitating PML^RING^ dimerization.[^87^, ^88^]

Interestingly, TRIM16, lacking the RING domain, retains E3 ligases activity which is attributable to its RING‐like folded B‐box domain.^[89]^ However, this is not a general feature, as in the case of TRIM66 loss of the RING domain correlates to the absence of ligase activity.^[90]^ Overall, these aspects in the TRIM protein family exhibit nice examples of structure activity relationships in multidomain proteins. However, only few studies on the molecular and biological functions of the B‐box domains have been reported so far, underscoring the need for further research on the topic.

### 3.4 Coiled‐coil domain

The N‐terminal domain and the C‐terminal SBD of TRIMs are separated by the coiled‐coil domain (Figure 2). This domain is well conserved across all TRIM proteins. TRIM proteins have two coiled‐coil domains separated by a helical segment. Each coiled‐coil has hairpin shaped subunits forming an elongated antiparallel dimer,^[75]^ involving mainly hydrophobic side chain interactions (Figure 3 IV). This domain is crucial towards homodimerization of TRIMs and as such regulates its biological activity.^[75]^ Additionally, the coiled‐coil domain has been described as a platform for macromolecular interactions, driving the recruitment of cellular partners modulated by specific environmental conditions.[^91^, ^92^]

The importance of the coiled‐coil domain is highlighted by the observation that viral proteins are able to bind this domain, preventing activation, substrate recruitment and/or imparting allosteric changes in the protein structure.[^93^, ^94^] For example, TRIM25 has been reported to synthesize K63 polyubiquitylation chains on retinoic acid inducible gene‐I protein (RIG‐I), also known as DDX58. This event enhances RIG‐I viral RNA sensing activity and induces Interferon (IFN) production. The crystal structure of TRIM25 coiled‐coil domain and the influenza A virus non‐structural protein 1 (NS1) shows how viruses can interact with TRIM25. The NS1 protein targets the coiled‐coil domain and allosterically inhibits the interaction between this domain and the PRYSPRY domain,[^93^, ^95^] thus downregulating IFN production. Surprisingly, unanchored K63 chains still form, which shows that this interaction might specifically affect substrate recruitment without affecting the RING‐mediated ligase activity.^[88]^

Hence, in depth investigation on this interaction might bring out a valuable targeting strategy for TRIM25^[93]^ and functional characterization of this domain might provide the opportunity to selectively target the oligomerization state occurring among different TRIM family members.

### 3.5 PRYSPRY domain

TRIM proteins belonging to class I and IV have a PRYSPRY domain at their C terminus (Figure 2).^[96]^ Usually, proteins with this domain are involved in cytokine signaling, innate immunity pathways and retroviral restriction.^[97]^ Prime examples here are TRIM25 in RIG‐I ubiquitylation^[16]^ and TRIM5α, TRIM20, TRIM1 in viral restriction.[^98^, ^99^]

The PRYSPRY domain has a jellyroll β‐sandwich tertiary conformation with a hydrophobic core (Figure 3 V A).^[97]^ Sequence alignment studies directed on PRYSPRY domain containing‐proteins, including TRIM21 (PDB ID: 2VOK), TRIM25 (PDB ID: 4B8E), TRIM72 (PDB ID: 3KB5), revealed sequence conservation for structural elements such as β‐strands and α‐helices, but also conservation within loop regions, predicted to form the rim of the binding pocket.^[100]^ Alanine‐scan and mutational studies allowed the identification of conserved hot‐spot residues within these loop regions capable of π‐stacking interactions and polar H‐bonding.^[101]^ Despite these structural similarities, conservation of the binding mode was not reported among PRYSPRY‐containing TRIMs.^[101]^ Intriguingly, peculiar structural features predicted to direct the specificity of substrate recruitment, were also identified. In TRIM25, for example, three highly solvent‐exposed Phenylalanine residues, sitting above a motif capable of π‐stacking (W−R−R−W), uniquely characterize the base of the putative binding site.^[100]^ Immunoprecipitation studies suggested that the PRYSPRY domain of TRIM21 binds to the Fc fragment of IgG.^[102]^ Trowsdale and co‐workers crystallized this interaction (PDB ID: 2IWG), revealing a dimeric stoichiometry (TRIM21/Fc 2 : 1) of the complex. This structural module has been described as a modular scaffolding platform with antibody‐like variable loops, revealing the presence of two discrete binding sites accommodating the target protein, encoded by the PRY and SPRY subdomains, respectively.^[101]^ In accordance with this observation, mutational studies directed on TRIM25 revealed the importance of two key residues potentially involved in substrate recruitment: Asp488 and Trp621, located on the PRY and SPRY domains, respectively.^[100]^

Furthermore, co‐immunoprecipitation studies to investigate possible interaction between TRIM25 and RIG‐I, identified a putative second binding site on TRIM25, consisting of a hydrophobic patch hidden beneath the N‐terminal α1‐helix connecting the coiled‐coil and PRYSPRY B30.2 domain. Intriguingly, a potential interactive interface on RIG‐I for TRIM25 binding has been identified and it is expected to be activated by additional cellular stimuli (e. g., phosphorylation, RNA binding) to displace the α1‐helix and ultimately reach TRIM25 PRYSPRY binding pocket.^[103]^ Nevertheless, the precise nature of TRIM25/RIG‐I interaction remains unclear and a minimal recognition sequence on RIG‐I responsible for TRIM25 binding was not identified.^[104]^ A recent study reported that the PRYSPRY domain of TRIM25 mediates RNA binding.^[105]^ It has been suggested that this interaction might enhance ubiquitylation activity by facilitating substrate recruitment through binding to the same RNA.^[106]^ This aspect could explain why there is no clear evidence for a direct interaction *in vitro* between TRIM25 and RIG‐I. It might be interesting to investigate whether the RNA binding capacity is a peculiarity of TRIM25‐PRYSPRY or if it is a conserved feature across PRYSPRY‐containing TRIM.[^93^, ^95^]

The identification of potential ligand binding pockets with related hot‐spot residues capable of both hydrophobic and polar interactions, makes the PRYSPRY domain a promising candidate for the design of inhibitors targeting the C‐terminal domain‐mediated substrate recruitment in TRIM proteins.

### 3.6 PHD finger bromodomain

TRIM proteins belonging to class VI are also known as the transcriptional intermediary factor 1 (TIF1) family. In humans, this family comprises four proteins, TIF1α/TRIM24, TIF1β/TRIM28/KAP‐1, TIF1γ/TRIM33/Ectodermin and TIF1δ/TRIM66. Whilst TRIM66 lacks E3 ligase activity due to the absence of the RING domain, all members of this family own a plant homeodomain and bromodomain (PHD–BRD) motif at their C terminus (Figure 2). Structurally, the PHD is a zinc finger with two β‐turns coordinating with a zinc ion in a cross‐brace conformation (Figure 3 VI A).^[107]^ BRDs share a conserved overall fold comprising a unique left‐handed bundle consisting of four α helices (αZ, αA, αB, αC) linked by highly variable loop regions (ZA and BC loops) that form the docking site for interacting recognition motifs (Figure 3 VI A). Tandem PHD‐BRDs are often found in chromatin‐associated proteins and have been shown to cooperate in gene regulation with different mechanisms, as transcriptional activators or repressors.^[108]^ They specifically recognize combinations of PTMs on histones or other nuclear proteins, mainly represented by methylation and/or acetylation of Lys residues that together build up the epigenetic code. Some BRD‐containing TRIMs act as readers of this epigenetic code. For example, TRIM24 BRD has been described as dual reader that specifically recognizes H3‐K23^Ac^ and H3‐K4^Me0^.^[109]^ PHD‐BRD binding to chromatin in TRIM proteins can trigger ubiquitylation of transcriptional factors, linking the E3 ligase activity to the epigenetic regulation. In line with this, it is postulated that TRIM33‐mediated ubiquitylation of SMAD4 is activated only upon binding of TRIM33^PHD‐BRD^ to chromatin, in response to TGF‐β signaling.^[78]^ Furthermore, TRIM proteins can regulate transcription with mechanisms that do not imply histone binding. Accordingly, the PHD finger of TRIM28 lacks conserved residues normally directing histone recognition;^[110]^ thereby, it is considered a non‐histone binding epigenetic module. Conversely, TRIM28 promotes SUMOylation of the adjacent BRD, contributing to the regulation of the nucleosome remodeling and deacetylase (NuRD) complex.^[111]^

There are 61 human BRDs present in 46 different proteins, which are clustered into eight families (Figure 3 VI B), based on structure/sequence similarity.^[112]^ Moreover, BRDs belonging to family II are also known as bromo and extra‐terminal domain (BET). BET family members have been subject of intensive drug discovery efforts, resulting in effective anticancer and anti‐inflammatory strategies,^[113]^ with a number of inhibitors in phase I clinical trials.^[114]^ In contrast, non‐BET BRDs inhibitors have been developed to a lesser extent and none of them showed the sub‐micromolar inhibition that have been reported for BET BRD ligands.^[115]^ The comparatively high druggability of BET BRDs can be explained by the larger upper part of the K^Ac^ binding pocket and the longer ZA loop, providing additional surface that can be utilized for interactions with small molecules.

TRIM proteins are all classified as non‐BET BRDs and belong to different BRD clusters (class V and VI; Figure 3 VI B). Structural and functional characterization of PHD‐BRD motifs in TRIMs, along with the identification of specific binding partners, would help to elucidate their roles in chromatin regulation and/or in the ubiquitylation context.

### 3.7 NHL repeats

TRIM proteins belonging to class VII are characterized by the NHL repeats at their C terminus (Figure 2), associated with the Filamin‐type immunoglobulin domain (FN3; Figure 3 VII A). The NHL domain is roughly forty residues in length, arranged in four to six symmetrical blade‐shaped β‐sheets.[^28^, ^72^] The overall tertiary conformation results in a β‐propeller barrel with a canonical toroidal shape that enclose a central channel, usually funnel‐shaped (Figure 3 VII B).^[116]^ However, in some cases, a shallow pocket is observed instead of a well‐defined central channel.^[116]^ The NHL domain can mediate protein‐protein (substrate) interactions or protein‐RNA interactions. Several NHL‐containing TRIMs have been identified as part of the human RNA interactome, including TRIM2, TRIM3, TRIM32, TRIM56, TRIM71.^[117]^

The canonical NHL domains have a top and a bottom binding interface.^[118]^ All known interactions with RNA are mediated by the positively charged top surface.^[119]^ Their RNA‐binding capacity has important implications on NHL‐containing TRIMs activity either by facilitating substrate recruitment or by inducing allosteric changes in the TRIM protein to enhance ubiquitylation.[^119^, ^120^, ^121^] Despite the importance of this interaction, the involved interface on the NHL domain is highly polar with abundancy of positively charged residues. This aspect might limit the possibility of developing inhibitors with drug‐like properties targeting this domain. In addition, protein ligands including substrates can bind either to the top, the bottom surface or between two adjacent β‐propellers.^[116]^ Interestingly, the β‐propeller fold of the NHL repeats in TRIMs, predicted to act as SBD, is also found in WD‐ and Kelch domains of proteins acting as substrate receptors of CRL E3 ligases. For example, the WD repeat‐ containing proteins β‐TrCP and Cdc4 are substrate‐receptor regions of Skp1‐CUL1‐F‐box complexes,^[122]^ whereas Kelch domains recruit the substrate in BTB‐CUL3‐R‐box complexes.^[123]^ Moreover, the C‐terminal Kelch domain of Keap1, the substrate recognition component of BTB‐CUL3‐R‐box complex, has been crystallized bound to a peptide derived from its substrate: Nuclear transcriptional factor 2 (Nrf2). Characterization of this complex allowed for the identification of key interactions involved in substrate binding^[124]^ that have been exploited for the design of inhibitors targeting the multi subunit E3 CUL3^KEAP1^.^[125]^

In conclusion, the NHL domain in TRIM proteins has promising features that can be targeted for therapeutic application as inhibitors for their β‐propeller structural fold present in other proteins have successfully been developed,^[125]^ Unfortunately, no crystal structures for human NHL‐containing TRIM^FL^ or isolated TRIM^NHL^ domains have been reported so far, thereby limiting characterization efforts and computational studies necessary for drug development.

## 4 Tripartite Motif Proteins: Targeting Options

The consistent gap between structural features of TRIMs and precise molecular functions or biological outcomes relies on two main factors: the nonenzymatic scaffolding nature of these enzymes and the lack of known validated substrates targeted for ubiquitylation/SUMOylation/ISGylation (Table 2). These aspects limit the possibility of designing assays for compounds evaluation and inhibitor development. Our current understanding on the biological function of TRIM proteins is limited as it is represented by the variability of regulatory events directing their activity, including TRIM self‐interactions and oligomerization^[126]^ This complexity poses challenges in the definition of mechanistic models, experimental procedures and effective targeting options. The non‐enzymatic mechanism of Ub transfer makes TRIM ligases difficult to target with traditional small‐molecule ligands and conventional occupancy‐based methods. Nevertheless, their multidomain nature (Section3) which correlates with a multifunctional biological profile, might enable the design of alternative attractive targeting options. Indeed, addressing the activity of TRIMs is not limited to interfering with the E3 ligase activity, as these proteins can exert their cellular functions with a combination of E3‐dependent and independent mechanisms, including post‐translational modification of substrates, transcription regulation and interaction with cellular components (other proteins, RNA) or receptors.[^127^, ^128^] While this aspect increases the complexity of the target, it also provides a more valid rational for targeting as multiple strategies can be undertaken for the design of active compounds.

**Table 2:** Summary of proteins reported to interact with the C‐terminal substrate binding domain of TRIMs.

TRIM C‐terminal	Substrate/interactor
domains	
PRYSPRY	RIG‐I,^[75]^ MAVS,[^158^, ^159^] p65,[^160^, ^161^] TRAF2,^[161]^ TAK1,^[161]^ NEMO,^[16]^ TRAF3,^[160]^ RNA,[^119^, ^160^] ZAP[^105^, ^162^]
PHD‐BROMO	histone H4,^[163]^ Smad4,^[78]^ histone H3,^[78]^ β‐catenin,^[48]^ DHX33,^[164]^ HIV‐1 viral integrase^[165]^
NHL	mRNA,[^28^, ^116^] myosin‐V,[^28^, ^116^] miRNA302,[^28^, ^116^] miRNA290[^28^, ^116^]
MATH	MEKK4^[166]^
COS	microtubules and microtubule binding proteins^[167]^

**Figure 4:**
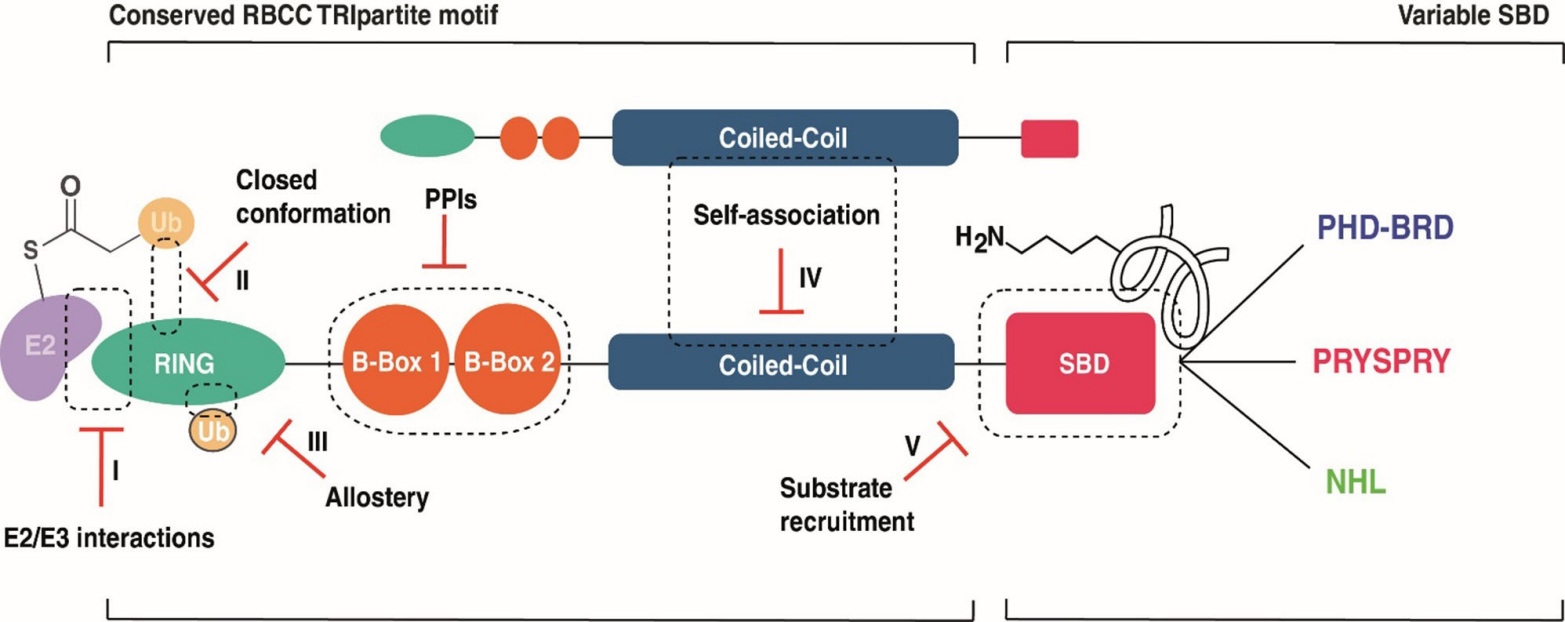
Schematic representation of potential targeting options for TRIM proteins. Inhibition strategies could be primarily achieved by targeting the N‐terminal conserved RBCC motif represented by I) inhibition of E2 binding; II) exploitation of the RING interactions with Ub to be transferred; III) exploration of the putative allosteric docking of unbound ubiquitin molecules; IV) interfering with coiled‐coil‐mediated self‐association or by targeting the variable C‐ter‐ minal‐mediated substrate binding domain (SBD). The most promising targeting strategy is represented by the latter (V). Inhibition of substrate recruitment with a special focus on three structural motifs found in TRIM proteins: PHD‐BRD cassette, PRYSPRY domains, and NHL repeats. Future research might also define a role and targeting mode for B‐box domain–mediated protein–protein interactions (PPIs).

As TRIM proteins function as scaffolds ensuring close proximity between the E2‐Ub conjugate and the substrate,^[15]^ inhibition strategies could be primarily achieved by targeting either the N‐terminal conserved RBCC motif or the C‐terminal‐mediated substrate binding domain (SBD; Figure 4).

### 4.1 Targeting the conserved TRIM^RBCC^

The conserved RBCC motif in TRIM proteins contains the functional unit responsible for E2 engagement (Figure 4, I) and catalytic transfer of Ub (RING; Figure 4, II) and the structural regions responsible for activation via self‐association (coiled‐coil mediated dimerization[^73^, ^75^, ^129^]) or oligomerization (higher order assembly via RING/B‐boxes[^80^, ^81^]), or heterotypic PPIs via coiled‐coil.[^91^, ^130^] Thereby, targeting of these domains has the potential of effectively and selectively affecting a precise event directing TRIMs’ catalytic activity. However, these inhibition strategies would require the development of PPI modulators. PPI inhibitors have been difficult to target in drug discovery, as the interaction interfaces are usually very large and contain many hydrophobic residues. Moreover, the peptide nature of potential drug candidates limits the achievement of drug‐like molecules with favourable ADME properties during the optimization phase of the drug discovery process. Nevertheless, substantial progress has been achieved during the last decade leading to several examples of peptidomimetic and small‐molecule PPI inhibitors that have been developed for other E3 ligases. The MDM2 inhibitors^[131]^ and selective ligands for the BIR domains of IAPs^[132]^ are illustrative to this extent.

#### 4.1.1 Targeting E2/E3RING interactions

Starting from the already available information on RING/E2‐Ub complexes,^[83]^ superposition studies among different TRIMs, supported by site specific mutagenesis experiments, may allow the identification of key pharmacophoric sites directing this macromolecular interaction. This approach has proven to be fruitful for TRAF6, sharing functional similarity to TRIM25. They are both single polypeptide RING E3 ligases that in presence of Ubc13/Uev1A, catalyze the K63‐linked polyubiquitylation of cellular propagators involved in autoimmune and inflammatory response.^[133]^ Co‐crystal structures of TRAF6/Ubc13, along with single point mutation studies, successfully demonstrated the importance of distinct residues of TRAF6 (Cys70, Asp57) in realizing the interaction with the Ub‐charged E2.^[134]^ This observation provided a rational basis for the design of small‐molecule inhibitors targeting Ubc13/TRAF6 interaction. In a later study, a small‐molecule PPI inhibitor of TRAF6/Ubc13‐Ub binding, named C25‐140, was found by the use of a high‐throughput screening (HTS) approach.^[135]^ C25‐140 resulted in improved outcomes of autoimmune psoriasis and rheumatoid arthritis in *in vivo* mouse models and, importantly, it is target‐specific and does not interfere with K48‐linked polyubiquitin chain formation, avoiding the risk of cellular protein homeostasis dysregulation.^[136]^

However, to pursue PPI modulators for TRIM family members additional studies are required. Crystal structures studying TRIM proteins are currently limited to subdomains of proteins in isolation and only in some cases to the co‐crystal structures of E3^RING^ : E2‐Ub complexes. Another important aspect to elucidate includes the identification of specific E2/E3 pairs for all TRIM proteins, which would require a systematic investigation. Additionally, the attribution of the specificity of the polyubiquitin chain linkages still remain enigmatic. The linkage specificity is mainly attributable to the E2 s, since some E3 s can bind different E2 s and catalyze different chain linkages (e. g., TRIM25/Ubc13: K63; TRIM25/UbcH5: K48). However, further studies are required to generalize and confirm this mechanism.^[15]^

#### 4.1.2 Manipulation by allostery

Allosteric regulation represents an important feature to explore in the case of TRIMs and RINGs, both to gain further insight into the regulatory events directing the ligases activity and as a strategy for the development of novel inhibitors. Allosteric inhibition of substrate recognition was successfully exploited for SCFCdc4, component of the E3 ubiquitin ligase complex SCF (SKP1‐CUL1‐F‐box protein), through the use of a small molecule, identified by HTS, that binds 25 Å away from the substrate binding site.^[137]^ More generally, there is evidence in literature supporting that not only E2 s and RING E3 s bind to promote ubiquitylation, but also the Ub to be transferred undergoes additional interactions with the RING domain in order to stabilize the closed conformation and enhance the efficiency of its transfer.^[53]^ In line with this observation, recent studies revealed distinct structural elements in RING E3 domains that recognize a consensus region on the Ub molecule to be transferred (Figure 4, II).[^82^, ^138^] Moreover, the E2 UbcH5 has been reported to possess a “backside” that supports interactions with a range of effectors including Ub and ancillary regions of monomeric RING E3 s to enhance affinity of the E3–E2 complex and allosterically regulate activity.[^139^, ^140^] When UbcH5 is loaded with a donor Ub, binding of a second copy of Ub to its backside markedly enhances the affinity of the conjugate to RNF38, thereby stimulating Ub transfer.^[141]^ Analyses of two related RINGs, Arkadia and Ark2C (Ark‐like RINGs) revealed that the RING domain itself can directly bind Ub, and that docking of the RING domain into a regulatory ubiquitin molecule promotes transfer of Ub to substrates.^[142]^

More specifically, the multidomain nature of TRIM proteins enables the occurrence of allosteric mechanisms of regulation directing their activity through self‐interactions of different functional domains present in the protein structure. In accordance with this observation, Koliopoulos et al. reported a weak interaction in solution between the coiled‐coil and the PRYSPRY domains of TRIM25. This intramolecular association is promoted by RNA binding and it is necessary for RIG‐I ubiquitylation.[^93^, ^95^] The importance of this regulatory mechanism is supported by the observation that the viral protein NS1 binds to the coiled‐coil domain of TRIM25 and allosterically inhibits its ubiquitylation activity.^[93]^

The presence of intra‐ and inter‐molecular mechanism of allosteric regulation in TRIM family members might represent a crucial aspect to elucidate and manipulate their activity (Figure 4, III).

#### 4.1.3 Interfering with dimerization and oligomerization

For the majority of RING ligases, RING domain dimerization is deemed to be required for activation and catalysis,^[73]^ providing the potential option of targeting dimeric TRIM ligases by PPI modulators preventing RING self‐association. The development of PPI inhibitors affecting homodimerization might target directly the RING or the coiled‐coil domain that is crucial in this process (Figure 4, IV). Additionally, several mechanisms of activation, including homotypic and heterotypic interactions involving the coiled‐coil, have been reported in the case of TRIMs.[^37^, ^126^, ^130^, ^143^, ^144^] The existence of specific mechanisms occurring in different TRIMs is predicted to direct the E3 ligase activity and promote substrate recruitment.

A successful drug development approach following this route would be limited by the extension of interactive interfaces and the required hydrophobicity for candidate active compounds that is difficult to combine with drug‐like properties. However, mutation studies directed on other RING ligases, demonstrated that two Val residues located in the RING domain of XIAP ligases are indispensable to allow dimerization;^[145]^ whilst structural analysis directed on MDM2 resulted in the importance of the C‐terminal amino acids for proper complex formation and stabilization of the overall RING structure.[^146^, ^147^] These examples suggest that despite extension of the involved interfaces, pharmacophoric sites can be selectively identified. Additionally, mutational studies directed on TRIM proteins demonstrated that disruption of the coiled‐coil domain dimerization relies on the presence of residues capable of H‐bonding rather than hydrophobic interactions.^[129]^ This aspect suggests that hydrophobic and hydrophilic requirements might be balanced for the design of potentially active compounds targeting the function of this domain.

### 4.2 Inhibition of substrate recruitment: targeting TRIM^SBD^

Substrate recognition is one of the most important steps in TRIM activity and usually, this aspect is mediated by its C‐terminal substrate binding domain (SBD). Although the biology aspects have been explored for many TRIMs, not much is known about their direct substrates. As targeting SBDs through competition of native substrates is the most common mechanism reported to inhibit RING E3 ligases, known interactors of different C‐terminal TRIM domains have been summarized in Table 2. It must be noted that distinction between interactors and substrates is challenging and strictly depends on the experimental procedure used. Hence, reported interacting partners might only be interactors instead of true substrates getting modified.

Inhibition approaches developed for IAP,^[69]^ MDM2^[68]^ and VHL^[67]^ are illustrative for this. Clinically evaluated IAPs antagonists^[148]^ were developed by utilization of the first four residues, AVPI (Ala, Val, Pro, Ile), of the N terminus of Smac/DIABLO, a natural antagonist of XIAP that binds to its BIR3 domain.^[149]^ In the case of MDM2 the interaction between its N terminus domain and the transactivation domain of p53, revealed the presence of two key hydrogen bonds and a triad of hydrophobic residues on p53 that crucially interacts with the binding cleft of MDM2.^[68]^ These features have been exploited for the development of peptidic and non‐peptidic modulators for MDM2/p53 PPI. Similarly, structural characterization of HIF‐1α/VHL interaction, led to the development of moderately potent inhibitors based on the presence of a key hydroxyproline residue from the HIF‐1α‐binding region.[^150^, ^151^, ^152^, ^153^] The above‐mentioned examples corroborate the importance of identifying the minimal structural requirements from the substrate that are responsible for E3 recognition and engagement.^[154]^ According to this, targeting of TRIM^SBD^/substrate interaction (Figure 4, V) would require significant efforts towards the discovery of specific endogenous substrates targeted for PTMs; hence, related crystal structures could be used as starting point for the development of peptide analogues or small‐molecule modulators. Importantly, knowledge on the substrate is crucial towards the design of appropriate screening assays and evaluation of potential inhibitors. The identification of substrates for TRIMs requires on one hand the use of advanced technologies to detect E3‐interacting proteins and, on the other hand, further validation of binders as *bona fide* substrates by orthogonal methods. Detecting substrates for E3 ligases is very challenging, due to the weak physical interaction and rapid dissociation rate between E3‐substrate complexes. Additionally, the low abundance of substrates targeted for proteasomal degradation, the redundancy of the system in which individual substrates can be targeted by several E3 s and the reversibility of ubiquitylation due to the coordination of ligases with DUBs activity, complicate this process.^[155]^ Thereby, integrated approaches combining genetic models with proteomics and molecular biology, are required to overcome these limitations, as discussed in Section5. Current efforts are mostly limited to computational approaches, including superposition studies,^[100]^ druggability analysis^[156]^ and *in silico* virtual screening.^[157]^ Based on structural and conformational analysis of TRIM^SBD^ and depending on the availability of crystal structures and chemical tools (including inhibitors, compound libraries, controls, assay platforms for activity and selectivity screening), the most promising candidates for the development of small‐molecule ligands include the PHD‐BRD cassette, the PRYSPRY domain and the NHL repeats.

#### 4.2.1 Targeting the PHD‐BRD domain

TRIM ligases belonging to class VI, owning a PHD‐BRD cassette at their C terminus, usually recognize combinations of PTMs on histones, including K^Me^ and K^Ac^. This aspect represents a remarkable advantage, since the K^Ac^ binding mode to the BRD is mostly conserved^[112]^ and K^Ac^ mimetic structures can be evaluated to design inhibitors.[^168^, ^169^] Generally, the bromodomain K^Ac^ binding pocket is of sufficient size to accommodate chemical inhibitors of 300–500 Da, in combination with their weak interaction with targeting sequences, make BRDs attractive sites for the development of inhibitors.^[112]^

Targeting of BRDs is supported by the development of efficient screening methods, including fragment‐based drug discovery (FBDD) that makes use of low molecular weight molecules whose weak binding to the target protein can be evaluated by using highly sensitive methods of detection (NMR, SPR). This strategy might be of particular interest for TRIM proteins, since native binding partners or tool compounds are currently unknown. Although current clinical compounds did not originate from this approach, there are several examples of successful optimization of fragments into selective probe molecules targeting human BRDs, including BET^[170]^ and non‐BET BRDs.^[171]^ Although, the druggability of the PHD domain has not been subjected to a comparable systematic investigation, a recent study reported the first FBDD approach to identify small molecules that bind the PHD finger domain.^[172]^ This study demonstrated the potential of NMR‐based fragments screening to explore the ligandability of this epigenetic module. However, low solubility issues, low affinity (evaluated in terms of ligand efficiency LE) of candidate peptide‐like ligands and the occurrence of pseudoligand interactions, suggests that targeting the PHD compared to the BRD domain is more challenging.[^172^, ^173^]

**Figure 5:**
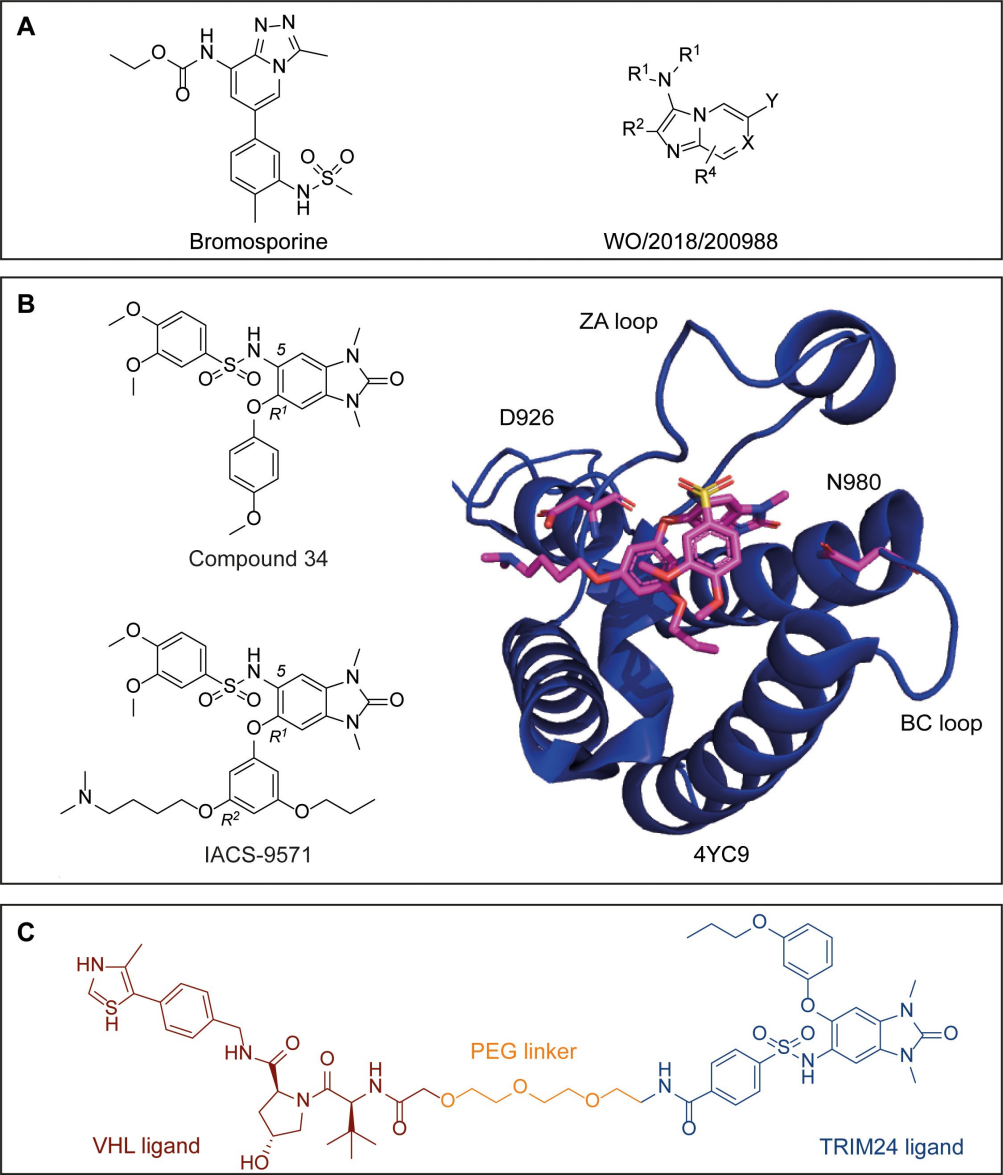
Published TRIM BRD inhibitors. A) Bromosporine, a broad‐spectrum BRD inhibitor (left); general formula of a patented TRIM33 BRD inhibitor (right); B) TRIM24 BRD inhibitors: Compound 34^[157]^ and IACS‐9571^[175]^ (left). Both compounds are based on a 1,3‐dimethylbenzimidazolone scaffold decorated with a sulfonamide in position 5 and a substituted phenyl in R^1^. In IACS‐9571 the introduction of a *N,N*‐dimethyl amino group on R^2^ with interposition of a short linker, promotes an additional ionic interaction; Co‐crystal structure of TRIM24 BRD and IACS‐9571^[175]^ (PDB ID: 4YC9) (right) showing the conserved folding of the BRD and the ligand interacting with two key residues: N980 (H‐bond between the K^Ac^ mimicry carbonyl moiety and the NH_2_ of the conserved Asn residue) in the BC loop and D926 (ionic interaction between the positively charged tertiary amine in R^2^ and the negatively charged carboxylic moiety of the Asp side chain) in the ZA loop. C) PROTAC for TRIM24 (dTrim24), made up of a PEG linker connecting a derivative of IACS‐9571 and a known VHL binder (VH032).

Very recently, a patent reporting the identification of a series of compounds as ligands for TRIM33 BRD was published (Figure 5A).^[174]^ However, TRIM33 exists as two isoforms (α and β), and the patent does not specify which isoform was used for compound evaluation. TRIM33α has a noncanonical BRD due to a 17 amino acids deletion. This feature might implicate challenges in the development of small‐molecule ligands for TRIM33α as well as opportunities to design structurally distinct and selective inhibitors over the two isoforms. Unfortunately, a detailed description concerning biophysical measurements and crystallography to establish binding mode, is not provided for the patented inhibitors.^[174]^ A recent study investigated the structure‐based druggability across BRD family members, thereby elaborating on a qualitative classification of human bromodomains according to unique signatures characterizing the binding pockets.^[156]^ All TRIM BRDs were predicted to be difficult to target, implying that they might show lower hit rates in screening efforts or limiting ligand optimization. Additionally, TRIM33 and TRIM24 share high sequence similarity, thereby posing challenges in the design of highly selective ligands. Nevertheless, recent work from the SGC and Bayer led to the development of dual BRPF‐1/TRIM24 BRD inhibitor, named Compound 34. It was obtained by screening of commercial 1,3‐dimethyl‐benzimidazolone scaffolds, known K^Ac^ mimicry, decorated with a sulfonamide in position 5 and a substituted phenyl in R^1^ (Figure 5B).^[157]^ In a different study, optimization of the same *N,N’*‐dimethyl‐benzimidazolone motif led to the discovery of IACS‐9571, by introduction of a *N,N’*‐dimethyl amino group on the aromatic ring in R^2^ and interposition of a short linker to promote a ionic interaction with D926^[175]^ (Figure 5B). The co‐crystal structure of this compound with TRIM24 PHD‐BRD revealed the expected globular domain organization of the BRD and the expected binding mode of the K^Ac^‐mimetic benzimidazolone moiety (Figure 5B). The activity of these inhibitors, tested on diverse cancer cell lines, did not give the expected antiproliferative effects or other related phenotypes, despite genetic lockdown of TRIM24 resulting in antiproliferative phenotypes and overexpression of TRIM24 correlated with poor patient prognosis.^[58]^ These findings suggest that TRIM^BRD^ inhibition alone might not be sufficient as an anti‐cancer strategy. However, the use of these compounds may prove therapeutically promising in combination with recently developed approaches, such as the PROTAC design, as described in Section 4.3.

#### 4.2.2 Targeting the PRYSPRY and NHL domain

Other C‐terminal SBDs that represent promising candidates for ligand development, include the PRYSPRY domain, present in the majority of TRIMs, and the NHL repeats, characterizing class VII (Figure 2). Both structural modules have been reported to mediate either PPI or noncoding RNA binding.[^95^, ^117^] The capability of NHL/PRYSPY‐containing TRIMs of recruiting substrates to be ubiquitylated and/or bind RNAs offers multiple possibilities to design inhibition strategies. However, the precise mechanism of these interactions remains unclear and requires further investigation. Additionally, it should be considered that candidate RNA‐mimic compounds might result extremely polar to be compatible with therapeutic application. Nevertheless, their use in *in vivo* and *in vitro* assays might help to elucidate the complex network linking RNA binding, ubiquitylation and innate immunity pathways. To this extent, a recent study reported the selection and *in vivo/in vitro* application of ssRNA aptamers specific to NS1 to rescue TRIM25‐mediated RIG‐I ubiquitylation from viral inhibition and repristinate IFN production.^[176]^ Although the precise mechanism determining this experimental outcome is not clear, this study offers a valid example of how the multidomain nature and multifunctional role of TRIMs can increase the number of possible targeting approaches.

For PRYSPRY‐containing TRIMs, two putative binding sites (located on the PRY and SPRY subdomains, respectively) have been identified in TRIM21 and TRIM25 that are predicted to mediate substrate recruitment.[^100^, ^101^] In particular, hot‐spot residues within this putative binding pockets are capable of both hydrophobic and polar interactions, increasing the probability of obtaining inhibitors with drug‐like properties.[^101^, ^103^] On the other hand, different studies reported the importance of the coiled‐coil domain and the involvement of an amino acid stretch in the PRY motif in realizing RNA binding.^[177]^ In addition, the linker connecting the PRYSPRY and coiled‐coil domains contains a short Lys‐rich motif that contributes towards this interaction.^[106]^

The β‐propeller tertiary conformation that characterizes the NHL repeats in TRIM proteins is also found in in WD and Kelch domains containing proteins that function as substrate‐receptors of CRL complexes. Importantly, these domains proved to be druggable in the context of multi‐subunit RING E3 ligases and inhibitors targeting this structural fold have been previously developed, as demonstrated by KEAP1/Nrf2 inhibitors.^[125]^ To date, no inhibitors have been reported for these structural modules present in TRIM proteins, as substrates targeted for PTMs are unknown. Moreover, the scarce availability of crystal structures for the human isoforms, either as full‐length proteins or isolated domains, represents a crucial limiting factor. Here, the availability of known substrates is of primary importance, as ligand/enzyme complexes are necessary to evaluate the conformational effects distinguishing the apo and bound structures of the TRIM protein.

In NHL domains, the RNA binding site has been associated to the positively charged top surface;^[117]^ however, the binding mode can vary among family members. For example, in BRAT the binding site is located on the top surface, whereas TRIM71 binds RNA through a positively charged shallow central cavity.[^119^, ^121^] As protein binding can occur on the top or the bottom surface of the NHL domain,^[117]^ whereas the RNA binding interface is generally represented by the top surface,^[117]^ it might be interesting to explore the mechanism of competitive binding/inhibition that can be realized when protein and RNA binding sites are overlapping.

Together, these considerations corroborate the potential of targeting the C terminus TRIM^SBD^; whilst emphasizing the imminent need of structurally and functionally characterizing TRIMs towards a rational for the design and evaluation of inhibitors.

### 4.3 Targeting autoubiquitylation

There are interesting regulatory events modulating the activity and stability of TRIMs that might be exploited for the design of indirect inhibition strategies. For example, autoubiquitylation or self‐ubiquitylation is an important process reported for some TRIMs.^[178]^ Autoubiquitylation was observed for the first time in TRIM5α, in both humans and rhesus monkey variants. Sequence alignments showed remarkable similarity between these two TRIM5α variants; however, they significantly differ in their antiviral potency in HIV‐1 restriction. Comparison of the autoubiquitylation potential among the two variants, in presence of suitable controls, demonstrated that rhesus monkey TRIM5α is substantially less self‐ubiquitylating than its human variant, and thereby more effective in HIV‐1 restriction. Although the type of linkage for ubiquitylation is unknown, this process may dictate huTRIM5α stability in cells, reducing its anti‐HIV activity. In light of this observation, autoubiquitylation might potentially be prevented or enhanced as indirect strategy to modulate the stability and activity of a specific disease‐related TRIM. It might be interesting to systematically explore this phenomena among members of the TRIM family.^[178]^

### 4.4 PROTACs

An emerging strategy to drive the activity of RING E3 ligases towards therapeutic application or as chemical biology tool for target validation is targeted protein degradation (TPD). This strategy exploits the natural UPS and the substrate specificity of E3 ligases for chemically induced degradation of a specific disease‐related target protein, without altering the global cellular proteome. TPD can be achieved with different modalities of targeting, either by the use of bifunctional heterodimeric ligands, the so‐called proteolysis targeting chimeras (PROTACs),^[179]^ or by monovalent molecular glues.^[180]^ The former are characterized by the presence of a linker connecting two different parts that specifically bind the protein of interest (POI) to be degraded and the E3 ligase, respectively.^[179]^ The latter are monovalent molecules that function as enhancers of E3/substrate PPIs.^[181]^ From the one hand, occupancy‐based inhibition methods imply the development of potent and selective small‐molecule ligands directed to the catalytic site of the protein of interest. On the other hand, protein degraders are recruiter molecules whose role is to ensure close spatial proximity between the E3 and the substrate, enhancing a naturally occurring cellular pathway that is represented by ubiquitylation, proteasomal recognition and subsequent degradation of the target protein.^[182]^ As a consequence, no functional activity towards the E3 or the substrate is required and even weak binders targeting a noncatalytic domain of the protein of interest can be promising candidates for TPD.^[183]^ Paradoxically, a tight binding might be disadvantageous in the case of protein degraders, as a long residence time (high‐affinity ligands) of the protein degrader in the E3/substrate complex would limit the number of target proteins that can be recruited by the degrader molecule. Through their unique mode of action, PROTACs have a completely new pharmacodynamic profile, allowing for higher potency at lower doses, prolonged pharmacological effect and decreased risk of off‐target binding and side‐effects.^[184]^ These features highlight the relevance of this targeting mode for multidomain proteins like TRIMs, thereby offering the possibility to expand the druggable space for scaffolding non‐enzymatic proteins like RING ligases.

The potential of PROTACs application has been expanded over the past few years through the use of PHOTAC and PhosphoPROTACs,^[185]^ where the ligand for the E3 recruitment is selectively activated by a sequence‐specific phosphorylation event or UV irradiation, respectively, thereby conferring spatiotemporal precision of targeting. Additionally, combined technologies making use of tagged proteins have been developed, as HALOtag^[186]^ or dTAG.^[187]^ However, despite the potential and versatility of PROTACs, only a few of the 600 family members of E3 s have been explored in targeted protein degradation to date, depending on the availability of ligands to engage the desired target protein and the required E3. PROTACs were initially developed for MDM2 and IAPs E3 ligases and the field remarkably accelerated when cullin‐RING E3 ligases were found to be addressed with small molecules, in particular CRBN and VHL.^[188]^ Recently, new E3 ligases have been reported to be used for protein degradation, including DCAF15,^[189]^ RNF114^[190]^ and RNF4.^[191]^

PROTACs have been successfully developed for BRDs, including human Bromodomains BRD4, belonging to the BET family^[192]^ and BRD9, a non‐BET BRD containing protein.[^193^, ^194^] A recent study by Bradner and co‐workers describes the first PROTAC targeting the bromodomain of a TRIM ligase.^[195]^ Here a derivative of the BRD inhibitor IACS‐9571 was exploited towards the design of a VHL‐based PROTAC targeting TRIM24 for proteasomal degradation (Figure 5C). The TRIM24‐based PROTAC (dTRIM24) was reported to drive potent and selective degradation of TRIM24 by recruiting VHL E3, resulting in inhibition of cell proliferation in acute leukemia cells. Interestingly, pairwise analysis of TRIM24 degradation versus BRD inhibition via IACS‐9571 alone revealed a beneficial selective action for the PROTAC approach.^[195]^

Although, Bradner and co‐workers described a PROTAC where TRIM24 embodies the POI to be targeted for degradation by CRL2^VHL^ E3; it would be interesting to explore the design of the reversed‐strategy degrader molecule as well. Here, TRIM24 could act as E3 ligase for protein degradation as it has been reported to negatively regulate the abundance of putative substrate proteins, such as p53, that are potentially targeted for K48 ubiquitylation by TRIM24.^[196]^ However, the existence of allosteric mechanisms of auto‐inhibition driven by self‐interaction of different functional domains present in the protein structure of TRIMs, might limit the application of this reversed approach.^[197]^

Given the potential of TPD approaches in revolutionizing the canonical way of targeting proteins, thereby expanding the number of possible druggable targets, research is currently pointing towards the identification of new ligands for other E3 ligases to enlarge the toolbox of protein degraders. It should be noted that size and polarity of PROTACs can pose challenges in terms of drug‐like properties; however, internal analysis suggest that the physicochemical properties of degrader compounds might be surprisingly more positive than expected in terms of cell permeability, systemic exposure and stability.^[184]^ These technologies have promoted a number of drug development research programs by pharmaceutical companies, resulting in several compounds, directed on either known or undisclosed targets, entering clinical testing in the past year with more to follow in the imminent future.^[198]^ Together these studies will reveal the true potential of TPD approaches.

## 5 Recent Advances To Explore the Ubiquitylation Machinery

Targeting the activity of E3 ligases represents the most specific and powerful way for therapeutic intervention within the ubiquitylation cascade. However, it comes with several challenges in the case of TRIMs, due to their scaffolding nature and scarcity of information with regard to biophysical and biochemical characterization of key macromolecular interactions. Multidisciplinary approaches are required to shed light on activity and functions of TRIM proteins.

Current available techniques in molecular and chemical biology, offer a variety of options to study the ubiquitylation machinery by combining genetics, functional genomics and proteomics approaches with chemical inhibition.^[199]^ Cellular and *in vivo* models that allow knock down or overexpression of virtually any gene have established the importance of TRIM proteins as targets for drug discovery.^[56]^ On the other hand, the identification of the precise biochemical linkage existing between ubiquitylation of substrates and the generation of physiologically altered cellular phenotypes is still poorly understood. To this extent, target validation is crucial to drive a successful drug discovery process. Advanced cellular systems to reconstruct the ubiquitylation cascade,^[200]^ the possibility of expressing affinity‐tagged enzymes (e. g., His tag) to facilitate purification and immunoblot analysis^[201]^ or the versatility of mass spectrometry (MS) tools to perform proteomic analysis and identify protein complexes in pull‐down experiments, supports this unmet need.^[202]^

In the case of TRIM ligases, E2 specific pairs or endogenous substrates are mostly unknown and the attempts to identify cellular interaction partners is limited by the fact that most of the above‐mentioned methodologies can be affected by several artifacts or are unable to detect weak complexes. For example, the attribution of enriched or depleted proteins as putative substrates for specific TRIM ligases in over‐expression based screens might be unreliable due to the redundancy of the ubiquitylation machinery. Nevertheless, recent advances in the field allowed to overcome this drawback, as in the employment of targets of ubiquitin ligases identified by proteomics 2 (TULIP2) methodology, which uses E3‐Ub fusion proteins to detect substrate interactions after purification of the His‐tagged trimeric complex E3‐Ub‐substrate.^[203]^ Furthermore, novel technologies for the detection of polyubiquitylated substrates by using ubiquitin binder sequences with dissociation constants in the nanomolar range, have been developed, as in the case of tandem ubiquitin binding entities (TUBEs); linear fusions of ubiquitin binding domains able to specifically bind tetrameric ubiquitin chains on target proteins.^[204]^

In addition, the development of *in vitro* reconstituted assays to demonstrate the ligase activity with possibility of chemically manipulating it by means of probes and inhibitors would represent the golden standard to identify the physicochemical events regulating enzyme activity. A chemical probe can be defined as an entity capable of binding to a given target with high *in vitro* potency (<100 nM, *K*_D_ or IC_50_), good selectivity (>30‐fold activity against other families) and evidence of cellular target engagement (compound concentration <1 μM).^[115]^ Advanced activity‐based probes (ABPs) undergo activity‐dependent labeling of enzyme family members and their use is very versatile. Ubiquitin‐based chemical probes have been successfully used for crystal structural studies, for protein profiling to study enzyme regulation or for inhibitors screenings.^[13]^ Traditionally, they have been developed for enzymes that target the catalytic Cys in E3 s or DUBs.^[205]^ In the case of scaffolding proteins like TRIMs, the absence of the catalytic Cys in the design of ABPs might be partially overcome by the incorporation of photocrosslinking moieties (e. g., Bpa: *p*‐benzoyl‐β‐phenylalanine) within the E2‐Ub conjugate. Such a strategy has been previously used for metalloenzymes^[206]^ and more recently for SUMO E3^[207]^ and RING E3 s.^[208]^

The design of a robust reconstituted *in vitro* assay to monitor the ubiquitylation of substrates, suitable for HTS would enable both mechanistic studies of Ub transfer as well as small‐molecule modulator screens for TRIM proteins. To this extent, a variety of synthetic and semisynthetic approaches to obtain Ub and UBL‐based reagents have been developed^[209]^ allowing for different assay readouts. Fluorescence polarization (FP)‐based screening methods have been successfully used to monitor DUBs activity, mainly employing Ub‐AMC or Ub‐rhodamine, which fluoresce only after cleavage.^[210]^ In the case of Ub conjugation, screening approaches are much less standardized, however simplified reconstitution systems have been reported in the case of HECT and RBR ligase.^[211]^ UbiReal was described as a simple HTS approach to track all stages of Ub conjugation and deconjugation in real time relying on the use of fluorescently labeled Rho‐Ub and implying a FP‐based readout.^[212]^ On the other hand, time‐resolved (TR) FRET assays represent a valuable approach to monitor RING‐mediated Ub‐chain conjugation and RING ligase activity. For example, the combination of TR‐FRET technology with TUBES *in vitro*, has successfully been employed to monitor autoubiquitylation of MuRF1 and TRIM25.^[213]^ In an attempt to study the activity of non‐enzymatic proteins like TRIMs and evaluate the effect of potential inhibitor candidates, commercially available assay platforms to detect PPIs might prove useful. Alphascreen makes use of fluorophore‐containing tagged donor and acceptor beads able to recruit the labeled enzyme and substrate, respectively, whose reduction in signal emission via FRET is used to evaluate binding of candidate inhibitors.^[214]^ Although susceptible to assay interference and prone to false positive results, it might represent a powerful high‐throughput assay for the initial screening of PPIs modulators. Additionally, the Alphascreen TruHits kit can be used to identify false positives resulting from singlet oxygen quenching or biotin mimetics.^[214]^ In the absence of ligands as starting point for inhibitors design and optimization, fragment‐based screening approaches might represent the most promising way to define potent and selective ligands for E3 ligases. With a robust *in vitro* assay suitable for HTS, the use of low‐molecular‐weight fragments allows sampling of a greater proportion of chemical space than an equivalent sized library of lead‐like molecules.^[215]^ Initial fragment hits can be developed into drug‐like molecules using techniques like fragment linking, merging, or growing, by preserving crucial parameters, like LE (ligand efficiency) and LEE (ligand lipophilic efficiency). Developing robust *in vitro* assays require extensive protein purification, laborious characterization of co‐factors, substrates and proper choice of a detection technology for the screening assays. Moreover, reconstituted activity assays do not take into account cellular context and localization of involved components. To address these limitations, cellular‐based screening technologies have recently been improved. The Ubiquitin Ligase Profiling platform (UPL) represents a cellular HTS system that combines TUBEs with the NanoBiT luminescence‐based technology to monitor ubiquitylation of E3 s and it was successfully used to evaluate the activity of inhibitors against RNF8 E3 ligase.^[216]^

Another promising strategy to address the activity of multidomain proteins, consists of exploiting the druggability of determined structural modules that have been successfully targeted, either to find a functional inhibitor or to identify a binder for PROTAC design. BRD‐containing TRIMs provide an illustrative example here. Isolated BRD can be expressed and properly folded separately from the rest of the BRD‐containing protein and, importantly, the development of molecules to target BRD is promoted by commercially available chemical tools, including control compounds, histones mimicry libraries and assay platforms suitable for HTS. Bromosporine is a pan‐ BRD inhibitor that canonically binds to the K^Ac^ binding pocket of the BRD (Figure 5A).^[217]^ BROMO*scan*, originally developed to assess selectivity among kinase inhibitors, measures the binding of DNA‐tagged bromodomain to an immobilized BRD ligand (K^Ac^‐containing histone mimic). If an inhibitor is present, it will compete with the bromodomain binding to the immobilized ligand, resulting in reduction of a quantitative PCR (qPCR) signal in a dose‐dependent manner.^[218]^ This method of assay readout is less prone to compound interference compared to other displacement assays, such as FP or FRET (Alphascreen). Additionally, the affinity and selectivity of inhibitors can be evaluated against a comprehensive set of recombinant human BRDs in label‐free methods, including NMR‐based approaches^[219]^ and Isothermal calorimetry (ITC) that can be used for the direct determination of binding constants (*K*_d_) in solution, providing information on enthalpic, entropic contribution and stoichiometry of ligand binding.^[220]^

## 6 Summary and Outlook

TRIM proteins represent a promising target for the development of novel therapeutics. However, despite the recent advances made in developing innovative reagents and methodologies to study the ubiquitin field, there are still many conundrums to be resolved regarding our current understanding on this family of enzymes and their related potential use as pharmaceuticals. The current platform of reagents and assay readout have the potential to accelerate drug discovery efforts, targeting all aspects of the ubiquitin cascade and important lessons can be learned from other classes of RING enzymes. Yet, the frontier of inhibitor development lies in the introduction of innovative technologies as TRIMs are represented by their intrinsic scaffolding nature that limits the development of covalent probes, inhibitors or standardized screening methods.

The absence of a catalytic site limits the application of high‐affinity (covalent) inhibitors and low‐affinity inhibitors at high concentration may cause unfavorable off‐target effects. Additionally, the multidomain nature/multifunctional biological role of TRIMs implies that blocking just one function or one domain might not be sufficient to obtain the desired therapeutic outcome. Therefore, TRIM proteins are eligible targets for the development of protein degraders (PROTACs). Designing a ligand for any druggable region of a TRIM domain, independently from its function, could provide access towards therapeutically active degraders.

Giving the scarcity of structural and functional characterization for TRIM family members and the absence of known active compounds addressing their activity, there is no starting point for the design of inhibitors for these proteins. Additionally, *de novo* drug discovery requires high cost and time consumption; therefore, one promising strategy that is currently gaining attention and that could be useful to overcome these limitations consists in the evaluation of off‐patented drugs. A recent study screened a panel of off‐patent quinoline drugs, normally used as antimalaria, antiprotozoal and antibacterial agents, to evaluate their potential for the treatment of MM. Interestingly, it was found that NXQ (nitroxoline) can suppress cell viability and induce apoptosis by downregulating TRIM25,^[221]^ whose role in cancer development through regulation of p53 stability was previously reported.^[222]^ However, the precise mechanism remains unclear, since it is unknown whether NXQ can influence TRIM25 transcription and/or expression or affect the ligase activity.

In summary, the main bottleneck is currently represented by the lack of structural information and the multiplicity of mechanism of regulation that may occur in different TRIMs, along with the need to identify their endogenous substrates. Thereby, further investigation is stringently needed to functionally characterize TRIM proteins in detail and in return address their activity with effective targeting options for pharmaceutical applications.

## Conflict of interest

The authors declare no conflict of interest.

## Biographical Information

*Francesca D'Amico obtained her MSc in medicinal chemistry with honors at the University of Naples Federico II under the supervision of Prof. P. Grieco in 2018. During her internship in solid‐state NMR spectroscopy, she joined the group of Dr. M. Weingarth at the Bijvoet Centre for Biomolecular Research. She is currently a doctoral candidate in chemical biology, as Marie Skłodowska‐Curie ESR. As a member of the TRIM‐NET project, her research focuses on the investigation of the activity of TRIM E3 ligases through the development of effective targeting strategies for drug discovery*.

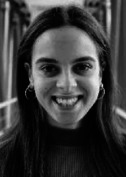

## Biographical Information

*Rishov Mukhopadhyay joined the laboratory of Prof. Huib Ovaa in 2019 to pursue a Ph.D. in chemical immunology under the ESR Marie Skłodowska‐Curie ITN: TRIM‐NET project. He obtained his BSc in pharmaceutical sciences from Jadavpur University (India) being awarded the University Gold Medal in 2014. He then moved to the University of Nottingham (U.K.) where he trained in structural biology under the supervision of Prof. J. Emsley and Dr. I. Dreveny, receiving his MSc in drug discovery and pharmaceutical sciences in 2017. His current research is focused on developing appropriate high‐throughput assays for TRIM proteins*.

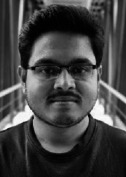

## Biographical Information

*On the 19th of May 2020, our dear colleague Prof. Dr. Huib Ovaa passed away from prostate cancer. He studied chemistry at Leiden University where he also obtained his PhD degree in organic synthesis in 2001 under the supervision of the late Prof. J.H. van Boom. He then moved to the lab of Prof. H. Ploegh at Harvard Medical School to become familiar with biochemistry and immunology. He moved to the Netherlands Cancer Institute in 2004 to start his own chemical biology lab. This lab was the basis of many ubiquitin chemistries used today in the construction of ubiquitin chains and reagents, proteasome technologies and antigen presentation. In 2010 he co‐founded the spinoff UbiQ Bio B.V. Since 2016 the lab has been at Leiden University Medical Centre and part of the newly created department of Cell and Chemical Biology. The lab focuses on basic discovery, probe and assay development, high‐throughput screening and medicinal chemistry*.

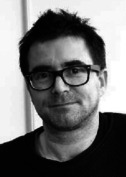

## Biographical Information

*Monique Mulder is assistant professor at the Department of Cell and Chemical Biology at LUMC. She received her MSc in chemical sciences with honours from Utrecht University in 2007, where she also obtained her PhD degree in medicinal chemistry in 2012 under the supervision of Prof. R.M.J. Liskamp. She then moved to the Ovaa lab at the Netherlands Cancer Institute. In 2016 she moved to the LUMC, where she is a Junior Group Leader. Her lab studies enzymatic action in the UPS using a combination of chemical synthesis, biochemistry and cell biology to discover unique components of the UPS that could become therapeutic targets*.